# Advancing Rehabilitation Medicine with the Metaverse: Opportunities and Challenges

**DOI:** 10.3390/brainsci15030321

**Published:** 2025-03-19

**Authors:** Rocco Salvatore Calabrò, Giovanni Morone

**Affiliations:** 1 IRCCS Centro Neurolesi “Bonino Pulejo”, 98123 Messina, Italy; 2 Department of Life, Health and Environmental Sciences, University of L’Aquila, 67100 L’Aquila, Italy; giovanni.morone@univaq.it

**Keywords:** metaverse, rehabilitation, virtual reality, augmented reality, stroke, function, participation, traumatic brain injury

## Abstract

The metaverse, an immersive virtual environment, is emerging as a transformative tool in rehabilitation, offering innovative modalities for motor and cognitive treatments. Virtual reality and augmented reality within the metaverse facilitate interactive exercises, allowing patients to perform rehabilitative tasks in a gamified context, which can improve motivation and adherence. Furthermore, the metaverse supports treatments that are not easy to carry out during conventional therapy, such as the rehabilitation of social participation, and creates a real individuals-based platform of continuum of care thanks to its interoperability. However, challenges such as technological accessibility, user adaptability, and the need for comprehensive clinical guidelines remain. Future research should focus on long-term efficacy, integration into traditional rehabilitation frameworks, and addressing ethical considerations, ultimately positioning the metaverse as a valuable adjunct in rehabilitative practices.

## 1. Introduction

The metaverse is a persistent, interconnected, and immersive digital ecosystem that integrates virtual and augmented environments, enabling real-time, multisensory interaction between users and artificial entities. The metaverse extends beyond traditional digital platforms by fostering a seamless convergence of physical and virtual realities, facilitating social, economic, and cultural activities within a shared, highly dynamic cyber-physical space [1]. This is possible thanks to the advanced and complex characteristics, including decentralization, interoperability, and scalability, leveraging advanced technologies such as artificial intelligence, blockchain, extended reality (XR), and high-speed networking.

Initially emerging as a gaming innovation, the metaverse is now envisioned as a transformative tool for transitioning from a traditional to a digital economy, attracting global stakeholders from various sectors. Moreover, because of the fusion of virtual and physical worlds, the metaverse promises a paradigm shift, particularly in the field of rehabilitation medicine [2].

Central to the metaverse’s operation are XR technologies, including virtual reality (VR), augmented reality (AR), and mixed reality (MR). While VR replicates the real world within a virtual environment, and AR overlays digital content onto physical spaces, XR technologies aim to deliver hybrid experiences that are interactive and immersive.

In the rehabilitation field, there is a substantial amount of literature demonstrating the effectiveness of VR and AR, as well as exergaming, in improving motor and cognitive outcomes in different neurological conditions [3].

It has been shown that the use of VR may be more effective than conventional physiotherapy in improving gait, balance, and non-motor outcomes, as well as quality of life, in patients with Multiple Sclerosis [4] and Stroke [5].

Concerning AR, a recent scoping review has demonstrated its potential in reducing disability and improving balance and gait outcomes [6].

Unlike VR and AR, which are mainly based on the simulation of immersive digital or hybrid environments, the metaverse represents a broader conceptual framework that integrates a service-oriented model with an emphasis on social interaction and digital content ecosystems. VR and AR technologies enhance sensory experiences by creating full virtual or augmented spaces. Then, they can be considered tools for specific use cases such as gaming, training, or remote collaboration. In contrast, the metaverse extends beyond these isolated experiences to create a persistent and interconnected digital environment where users engage with one another and with digital content in a dynamic and interactive manner [7].

A defining characteristic of the metaverse is its emphasis on user-driven interactions and content creation. Indeed, it supports a decentralized and participatory digital economy where individuals can create, own, and exchange digital assets, including virtual goods, services, and even property.

This perspective aims to explore the potential role of the metaverse in rehabilitation medicine, critically assessing its benefits and limitations in clinical practice. By leveraging insights from the relevant literature, we evaluated how the extended use of metaverse-based technologies may enhance patient recovery, optimize therapeutic interventions, and improve accessibility to rehabilitation services. Additionally, we discussed potential challenges, including ethical concerns, technological barriers, and clinical efficacy, to provide a balanced overview of the metaverse’s implications for future healthcare applications.

## 2. Toward the Use of Metaverse in Clinical Practice

VR and AR are specific technologies within the metaverse ecosystem. While VR and AR are typically applied for discrete experiences (VR as a completely immersive one and AR as enhancing the real world), the metaverse aims to bring these technologies together in a more integrated manner, where virtual worlds, data tracking, and simulations are combined into a seamless, immersive rehab experience.

In contrast to AR and VR, Lifelogging and Mirror Worlds offer the possibility of neurorehabilitation through the introduction of personalization and real-world simulation elements, enabling interventions that are not only immersive but also representative of the user’s own environment and progress. By incorporating these features, the metaverse offers a more interactive and dynamic neurorehabilitation process, allowing continuous interaction between the patient’s physical and virtual worlds, promoting engagement, and maximizing therapeutic effects [8].

Industries such as entertainment, tourism, and telemedicine have already adopted metaverse technologies, demonstrating their potential to integrate digital information seamlessly with physical environments [9]. In medical contexts, we foresee that the incorporation of metaverse technologies will overcome current challenges faced by VR solutions and boost rehabilitation therapy efficiency. With developments such as haptic feedback, interoceptive tech, and virtual avatars enabled by AI, the metaverse will enhance immersion therapy and provide modulation of clinical conditions including motor, pain, and psychiatric illnesses [10].

As recently highlighted, altering patients’ internal body representations through virtual embodiments has shown significant therapeutic potential [11]. Recent studies have demonstrated the importance of sensorimotor retraining, VR therapy, and brain–computer interfaces in recalibrating body schema to improve movement execution [11]. These innovative interventions engage body schema through immersive techniques, such as VR, which provides patients with real-time, interactive environments that facilitate sensorimotor integration and recovery. In this vein, the mirror neuron system (MNS) has emerged as a crucial neurophysiological mechanism underpinning motor rehabilitation recovery. Techniques such as action observation and motor imagery leverage MNS activation facilitate motor learning, even in the absence of voluntary movement [12].

Participation rehabilitation—helping individuals reintegrate into social and environmental contexts post-injury or illness—remains both the pinnacle goal and the Achilles’ heel of rehabilitation medicine. Standard approaches tend not to properly prepare patients for real-world interactions, particularly those who face social stigmatization or psychological barriers after events like brain injuries. For instance, stroke survivors who achieve physical mobility may still hesitate to re-engage socially due to fear of judgment. This disconnect represents not just a failure of rehabilitation but of healthcare [13]. The metaverse offers a unique solution to these challenges by enabling the simulation of real-world scenarios within controlled, immersive environments. Patients can engage in sensory, cognitive, behavioral, and motor activities designed to mirror their day-to-day lives, fostering confidence and adaptability. This aligns with the World Health Organization’s (WHO) International Classification of Functioning, Disability, and Health (ICF), which emphasizes a shift from pathology-focused care to person-centered functional recovery [13].

**Figure 1:**
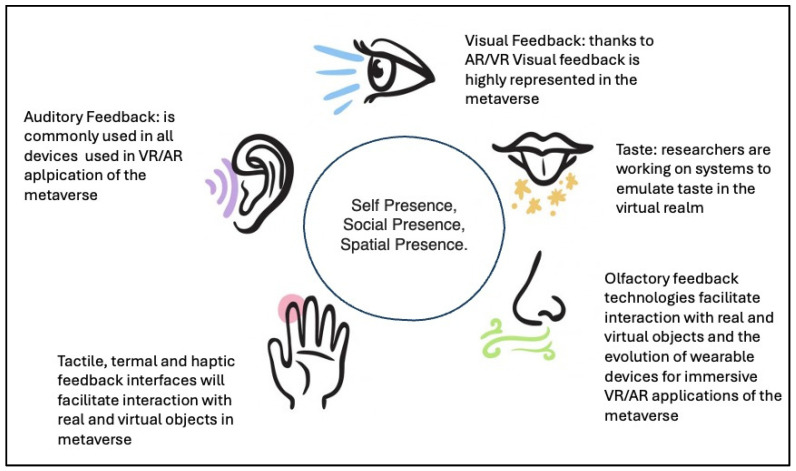
Representation of the senses involved in metaverse scenarios.

A compelling aspect of the metaverse is its capacity to integrate multisensory feedback into rehabilitation interventions. Haptic technologies, for instance, can simulate tactile sensations, allowing patients to practice fine motor skills or experience real-time feedback during virtual tasks. Similarly, auditory, visual, and more in general multisensory cues can be used to create immersive environments that replicate real-world challenges (Figure 1). These multisensory experiences not only enhance patient engagement but also promote neuroplasticity, a critical factor in recovery from neurological disorders [10,14].

The metaverse’s potential extends to addressing the cognitive and emotional aspects of rehabilitation, especially in patients recovering from traumatic brain injuries or stroke [15]. Virtual environments within the metaverse can be designed to target specific cognitive functions, such as memory, attention, and problem-solving, through gamified tasks and interactive and social challenges. Furthermore, the metaverse can provide a safe space for patients to confront and manage psychological challenges, such as social anxiety or post-traumatic stress disorder, by gradually exposing them to simulated social interactions [16].

The metaverse can support critical phases of rehabilitation, including prevention, continuity of care, education, and research. By harnessing emerging technologies such as holography and the Internet of Medical Things (IoMT), the metaverse can facilitate data-driven, AI-guided interventions for education, consultation, and treatment [17]. For example, patients could participate in collective “We-rehab” sessions, fostering social interaction and collaboration. Such collective training, as suggested by Riva et al., relies on shared goals and frames of reference, enhancing both motivation and adherence to therapeutic protocols [18].

Nonetheless, to date, only a few studies have properly used the metaverse as a potentially effective tool in improving rehabilitation outcomes. Moon et al. found that metaverse physical therapy improved gross motor function and cardiopulmonary function in patients with cerebral palsy and reduced the fear of getting COVID-19 [19].

It has been also shown that VR treatment delivered via the metaverse appeared to be safe (no adverse events or side effects) and led to a reduction of both low back and neck pain [20]. An ongoing study by Wu et is investigating the effect of a multimodal rehabilitation intervention on quality of life in patients with colorectal cancer survivors when delivered in the metaverse [16].

Moreover, the metaverse has also been recently used as a social VR-based collaborative exergame for the rehabilitation of elderly people. The participants found the social VR gameplay enjoyable and agreed that collaboration played a vital role in their motivation and reported various health benefits, with excellent usability scores [7].

## 3. The Use of the Metaverse for the Continuity of Care

In addition to enhancing rehabilitation participation, the metaverse has the potential to redefine the scope of care by making rehabilitation accessible beyond traditional clinical settings. With customizable environments, patients can engage in therapy sessions from their homes, reducing logistical barriers and improving continuity of care. This accessibility is particularly critical for patients in remote or underserved areas, where access to specialized rehabilitation facilities is limited.

**Figure 2:**
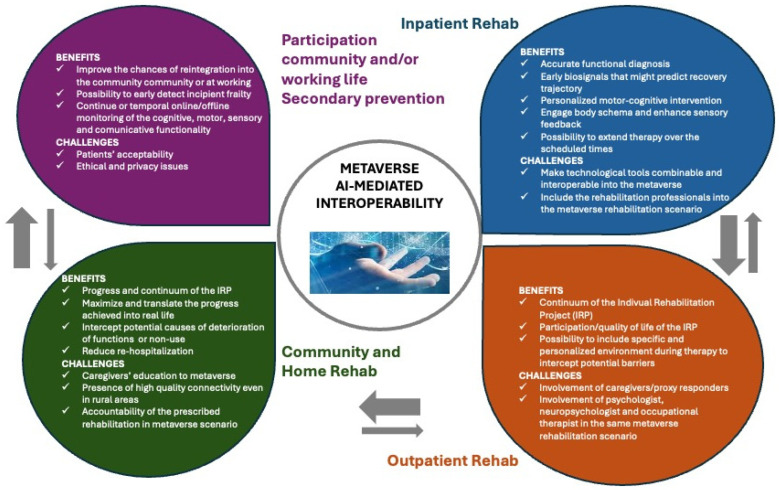
Schematic representation of the benefits and challenges of the rehabilitation continuum of care thanks to the feature of AI-mediated metaverse interoperability in different settings. The grey arrows indicate the flow of patients.

By spanning geographic and socioeconomic divides, the metaverse has the potential to democratize access to high-quality rehabilitation care so that no patient is left behind. Virtual clinics and tele-rehabilitation initiatives can deliver targeted, real-time interventions irrespective of a patient’s geographical location, bridging healthcare access disparities. Furthermore, immersive environments have the potential to support social support networks, increase patient engagement, and optimize treatment regimen compliance. By integrating AI-driven monitoring and real-time data sharing, healthcare providers can track progress more effectively, facilitating timely adjustments in care. Such a seamless, technology-supported process enhances continuity of care, allowing patients to receive consistent and integrated rehabilitation services regardless of physical constraints (Figure 2) [10,13,16].

## 4. The Metaverse Beyond the Patient’s Care

The implications of the metaverse for education and training in rehabilitation medicine are equally significant [21]. Healthcare professionals can use virtual platforms to simulate complex clinical scenarios, enhancing their diagnostic and therapeutic skills. For instance, therapists can practice advanced techniques in a risk-free virtual environment, ensuring that they are well-prepared to handle real-world cases. Additionally, the metaverse can facilitate interdisciplinary collaboration by enabling professionals from different specialties to interact and exchange knowledge in a shared virtual space [21].

From a research perspective, the metaverse offers a novel platform for conducting clinical trials and observational studies. By leveraging the vast amounts of data generated within virtual environments, researchers can gain deeper insights into patient behavior, treatment efficacy, and recovery trajectories [22]. This data-driven approach can accelerate the development of evidence-based interventions and inform best practices in rehabilitation medicine. However, it is crucial to establish robust frameworks for data governance to ensure ethical use and protect patient privacy [23].

## 5. Challenge and Limitation of the Metaverse

Despite its promise, integrating the metaverse into rehabilitation poses several challenges that must be addressed to realize its full potential and ensure its widespread adoption (see Table 1).

The metaverse can transform digital interactions, yet there are a few hurdles to be overcome if it is to lift off and be widely adopted. There are technical challenges, like providing interoperability across platforms and devices, which is one of the biggest challenges in creating metaverses [24]. The fact that there are no common standards for virtual environments, digital products, and user experiences leads to fragmentation, and companies and consumers cannot easily function on multiple metaverse platforms. Potential solutions include the development of open standards by groups like the Open Metaverse Interoperability (OMI) group and the Metaverse Standards Forum, blockchain-based asset portability adoption, and standardized API integrations for facilitating seamless interactions. Furthermore, the costly nature of developing and maintaining metaverse technologies can limit their utilization, particularly in low-resource settings. Requirements for infrastructure, like high-performance computing, cloud services, and VR/AR hardware, present significant financial barriers. Suggested remedies are harnessing open-source and affordable development tools like Mozilla Hubs and Unity’s OpenXR effort, stimulating government and institutional backing by public–private partnerships and grant programs, and using edge computing and cloud-based VR services to reduce the need for demanding local hardware [25]. Also, the metaverse entails pervasive data harvesting, comprising biometrics, user behavior, and transaction history, which portends grave privacy issues.

With weak security controls, the users are vulnerable to cyber-attacks, data breaches, and identity theft. Some of the potential solutions include blockchain-based decentralized identity systems, cryptographic technologies such as zero-knowledge proofs (ZKPs) for privacy protection, and the implementation of more robust regulatory frameworks modeled on global data protection standards like the EU’s GDPR. These will need to be tackled by collective efforts from industry stakeholders, policymakers, and researchers towards an open, secure, and interoperable metaverse economy that provides fair access and long-term sustainability [26].

Furthermore, the governance of digital health technologies should be guided by public interest rather than profit motives, ensuring that the metaverse serves as a tool for improving patient outcomes rather than a means for exploitation.

The ethical concerns surrounding patient data privacy in the metaverse are paramount, given the highly sensitive nature of personal health information (PHI) and the increasing integration of virtual healthcare environments. As telemedicine, virtual consultations, and AI-driven diagnostics become more prevalent in metaverse applications, robust safeguards must be established to prevent data breaches, unauthorized access, and misuse of medical records [27]. To mitigate these risks, regulatory frameworks must prioritize transparency, accountability, and patient autonomy. Existing regulatory frameworks such as the Health Insurance Portability and Accountability Act (HIPAA) in the United States, the General Data Protection Regulation (GDPR) in the European Union, and the Personal Information Protection and Electronic Documents Act (PIPEDA) in Canada provide crucial guidelines for ensuring data security and patient confidentiality. However, the decentralized and borderless nature of the metaverse introduces complexities that require adaptive policies and technological innovations. Implementing decentralized identity solutions using blockchain, cryptographic techniques like zero-knowledge proofs (ZKPs), and stringent regulatory oversight can enhance privacy protection while enabling seamless healthcare experiences in virtual spaces. Case studies such as VR for stroke recovery, AR for Alzheimer’s cognitive rehabilitation, Lifelogging in MS, and Mirror Worlds for SCI recovery demonstrate their effectiveness in improving patient outcomes. These technologies are regulated under frameworks like HIPAA and GDPR, ensuring data privacy and security. Together, they create an integrated, adaptive approach to neurorehabilitation, with strong regulatory oversight ensuring patient safety [10].

Ethical considerations must also address informed consent, data ownership, and the potential for algorithmic biases in AI-driven healthcare services, ensuring that patient rights remain safeguarded in an increasingly digitalized medical landscape.

Whereas the strengths of rehabilitation through the metaverse, e.g., more engagement and access from distant places, are known, the prospective weaknesses need to be noted as well to have an impartial debate. Prolonged activity in virtual environments contributes to mental consequences, i.e., fatigue from the digital world, dissociation, or demotivation for interaction in real life. Furthermore, metaverse-based rehabilitation remains an issue for impoverished patients who cannot afford VR hardware, high-speed internet, or compatible devices. Solutions to these issues involve special interventions like subsidized technology initiatives, the creation of lightweight VR alternatives, and hybrid rehabilitation paradigms that combine virtual and conventional therapy modalities. Moreover, questions regarding the efficacy of metaverse-based rehabilitation compared to conventional techniques must be alleviated through longitudinal studies and clinical trials to ascertain its long-term advantages. The inclusion of counterarguments and consideration of these challenges will ensure a more balanced and critically examined discussion of metaverse-based healthcare solutions.

## 6. Implementation of Metaverse-Based Rehabilitation in Clinical Practice

**Figure 3:**
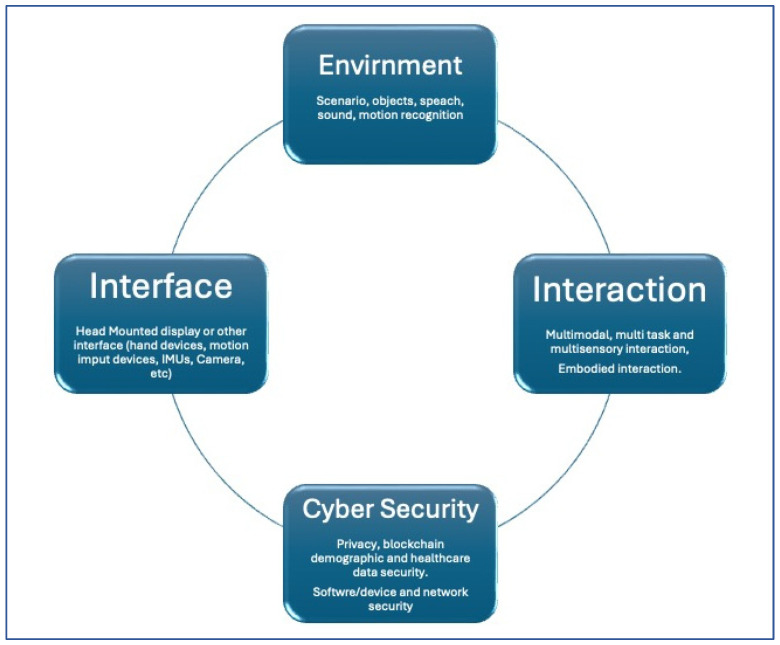
Metaverse and cyber security adapted for rehabilitation medicine.

Adoption of metaverse-based rehabilitation into clinical practice requires a systematic and evidence-based HTA, Health Technology Assessment approach that integrates it into existing rehabilitation procedures [28] and a solid cyber security operational framework adapted for rehabilitation medicine (Figure 3) [29].

Virtual therapy sessions represent one such approach, where patients receive supervised exercises in interactive VR environments. For example, survivors of stroke can receive gamified rehabilitation exercises that improve motor functions and neuroplasticity under real-time observation by remote physiotherapists. Similarly, cognitive rehabilitation of traumatic brain injury patients can be achieved through AI-driven virtual simulation that adapts in real-time to patient improvement. Metaverse platforms can be utilized for multidisciplinary team meetings, where rehabilitation specialists, neurologists, and psychologists can jointly assess patient improvement and make corresponding treatment plan modifications. In addition, virtual clinics can enhance accessibility by providing real-time feedback and monitoring with wearable biosensors and motion-tracking technology, with data-driven program modifications. To ensure successful implementation, healthcare institutions must develop training programs for clinicians to familiarize them with metaverse-based tools, as well as develop standardized protocols that are compatible with existing rehabilitation guidelines. Furthermore, reimbursement plans must be redesigned for payment for virtual rehabilitation services in insurance networks so that all patients, irrespective of socioeconomic status, have access on an equal basis. Pilot studies and clinical trials will be critical in establishing the efficacy of metaverse therapy and its integration into standard healthcare processes. Virtual therapy integrated with standard therapy will allow clinicians to provide more adaptive, interactive, and data-driven treatment sessions that maximize outcomes.

Interestingly, new hybrid stimulation, combining metaverse-based and real-world elements, might soon be challenging. Individuals might interact in a more enriched environment made by the presence of real objects (or devices) or real scenarios with modified characteristics and/or elements made by VR/AR [9,30]. A combined approach by VR/exergaming and noninvasive neuromodulation may further enhance both motor and cognitive outcomes, and they can pave the way for a potentiation/transformation of the treatment also in the metaverse environment [31]. In fact, similarly to the real environment, the combination of more technology-based intervention in the same therapy might enrich the rehabilitation, triggering more objective-ensuring fundamental principles to push neuroplasticity such as intensity, task specificity, salience, attentional involvement, motivation/participation, and novelty [32]. In this scenario’s hybrid approach, in the future, we might combine, for example, robots, treadmills with body weight support or sensor-based devices, and the metaverse, making possible the adaptation of the VR/AR context in a wide range of individuals’ functionality and not more for less-affected subjects.

The cultural shift required to embrace the metaverse in rehabilitation medicine should not be underestimated. Both patients and healthcare professionals may need time to adapt to this new paradigm. Educational initiatives aimed at increasing digital literacy and fostering acceptance of virtual technologies will be crucial for a smooth transition. By addressing these challenges proactively, we can create an ecosystem that supports the seamless integration of the metaverse into rehabilitation practice.

## 7. Conclusions

In conclusion, the metaverse represents a transformative opportunity to reimagine rehabilitation medicine, offering innovative solutions for patient engagement, functional recovery, and social reintegration. Its capacity to deliver personalized, accessible, and effective interventions has the potential to revolutionize healthcare delivery. By fostering interdisciplinary collaboration among researchers, clinicians, and technology developers, we can unlock the full potential of the metaverse to enhance patient outcomes and advance the field of rehabilitation medicine. However, its integration must be approached with caution, guided by robust ethical frameworks and substantial research investment. The metaverse is not merely a technological innovation but a tool to redefine the principles and practices of rehabilitation, paving the way for a more inclusive and effective healthcare future.

## Figures and Tables

**Table 1:** Opportunities and challenges of different metaverse aspects.

Aspect	Opportunities	Challenges
Social Interaction	Enhanced virtual communication, global connectivity, and new forms of socialization	Risk of digital addiction, loss of real-world social skills, and cyberbullying
Education and Training	Immersive learning experiences, real-time simulations, and global accessibility	Digital divide, high infrastructure costs, and potential misinformation
Healthcare	Virtual consultations, therapy in VR, medical simulations for training	Ethical concerns, data security, and need for regulatory frameworks
Business and Economy	Virtual marketplaces, remote work efficiency, and new economic models (NFTs, crypto)	Regulatory uncertainty, economic disparity, and cybersecurity risks
Entertainment and Media	Interactive storytelling, next-gen gaming, and live virtual events	Copyright issues, deepfake manipulation, and monetization challenges
Privacy and Security	Potential for decentralized, user-controlled data management	Data breaches, surveillance risks, and identity theft concerns
Inclusivity and Accessibility	Opportunities for disabled users, gender-neutral interactions, and broader access	Digital exclusion due to cost, hardware limitations, and accessibility gaps
Environmental Impact	Reduced travel emissions through virtual work and events	High energy consumption, e-waste, and carbon footprint of data centers
Ethics and Governance	New governance models, decentralized decision-making	Lack of legal frameworks, governance disputes, and content moderation issues

## References

[1] Morone G., Ciancarelli I., Calabrò R.S., Cerasa A., Iosa M., Gimigliano F. (2025). MetaRehabVerse: The Great Opportunity to Put the Person’s Functioning and Participation at the Center of Healthcare. Neurorehabilit. Neural Repair.

[2] Dong S., Liu M., Abbas K. (2024). The Metaverse Review: Exploring the Boundless Ream of Digital Reality. Comput. Mater. Contin..

[3] Maggio M.G., Maresca G., De Luca R., Stagnitti M.C., Porcari B., Ferrera M.C., Galletti F., Casella C., Manuli A., Calabrò R.S. (2019). The Growing Use of Virtual Reality in Cognitive Rehabilitation: Fact, Fake or Vision? A Scoping Review. J. Natl. Med. Assoc..

[4] De Keersmaecker E., Guida S., Denissen S., Dewolf L., Nagels G., Jansen B., Beckwée D., Swinnen E. (2025). Virtual reality for multiple sclerosis rehabilitation. Cochrane Database Syst. Rev..

[5] Wang S., Meng H., Zhang Y., Mao J., Zhang C., Qian C., Ma Y., Guo L. (2024). Effect of Virtual Reality-Based Rehabilitation on Mental Health and Quality of Life of Stroke Patients: A Systematic Review and Meta-analysis of Randomized Controlled Trials. Arch. Phys. Med. Rehabil..

[6] Hsu P.Y., Singer J., Keysor J.J. (2024). The evolution of augmented reality to augment physical therapy: A scoping review. J. Rehabil. Assist. Technol. Eng..

[7] Shah S.H.H., Karlsen A.S.T., Solberg M., Hameed I.A. (2022). A social VR-based collaborative exergame for rehabilitation: Codesign, development and user study. Virtual Real..

[8] Smart J., Cascio J., Paffendorf J., Bridges C., Hummel J., Hursthouse J., Moss R. (2007). A cross-industry public foresight project. Proc. Metaverse Roadmap Pathways 3DWeb.

[9] Dwivedi Y.K., Hughes L., Baabdullah A.M., Ribeiro-Navarrete S., Giannakis M., Al-Debei M.M., Dennehy D., Metri B., Buhalis D., Wamba S.F. (2022). Metaverse beyond the hype: Multidisciplinary perspectives on emerging challenges, opportunities, and agenda for research, practice and policy. Int. J. Inf. Manag..

[10] Calabrò R.S., Cerasa A., Ciancarelli I., Pignolo L., Tonin P., Iosa M., Morone G. (2022). The Arrival of the Metaverse in Neurorehabilitation: Fact, Fake or Vision?. Biomedicines.

[11] Riva G., Wiederhold B.K., Mantovani F. (2019). Neuroscience of virtual reality: From virtual exposure to embodied medicine. Cyberpsychology Behav. Soc. Netw..

[12] Calabrò R.S., Naro A., Russo M., Leo A., De Luca R., Balletta T., Buda A., La Rosa G., Bramanti A., Bramanti P. (2017). The role of virtual reality in improving motor performance as revealed by EEG: A randomized clinical trial. J. Neuroeng. Rehabil..

[13] World Health Organization (2001). International Classification of Functioning, Disability and Health (ICF).

[14] Morone G., Ghanbari Ghooshchy S., Palomba A., Baricich A., Santamato A., Ciritella C., Ciancarelli I., Molteni F., Gimigliano F., Iolascon G. (2021). Differentiation among bio- and augmented- feedback in technologically assisted rehabilitation. Expert Rev. Med. Devices.

[15] Cerasa A., Gaggioli A., Pioggia G., Riva G. (2024). Metaverse in Mental Health: The Beginning of a Long History. Curr. Psychiatry Rep..

[16] Hu Y., Peng H., Su G., Chen B., Yang Z., Ye Y., Zhou B., Lin S., Deng H., Zhang J. (2024). Effect of a metaverse multimodal rehabilitation intervention on quality of life and fear of recurrence in patients with colorectal cancer survivors: A randomized controlled study protocol. Digit. Health.

[17] Kaur J., Mehta S., Gupta S., Aljohani A., Khayyat M. (2024). Metaverse Medicine: Bridging Healthcare Gaps with AI and IoT Solutions. Impact and Potential of Machine Learning in the Metaverse.

[18] Riva G., Wiederhold B.K., Mantovani F. (2024). Searching for the metaverse: Neuroscience of physical and digital communities. Cyberpsychology Behav. Soc. Netw..

[19] Moon I., An Y., Min S., Park C. (2023). Therapeutic Effects of Metaverse Rehabilitation for Cerebral Palsy: A Randomized Controlled Trial. Int. J. Environ. Res. Public Health.

[20] Orr E., Arbel T., Levy M., Sela Y., Weissberger O., Liran O., Lewis J. (2023). Virtual reality in the management of patients with low back and neck pain: A retrospective analysis of 82 people treated solely in the metaverse. Arch. Physiother..

[21] Almeman K., EL Ayeb F., Berrima M., Issaoui B., Morsy H. (2025). The Integration of AI and Metaverse in Education: A Systematic Literature Review. Appl. Sci..

[22] Suh I., McKinney T., Siu K.-C. (2023). Current Perspective of Metaverse Application in Medical Education, Research and Patient Care. Virtual Worlds.

[23] Ling A., Butakov S. (2024). Trust Framework for Self-Sovereign ID in Metaverse Health Care Applications. Data Sci. Manag..

[24] Mohammadzadeh Z., Shokri M., Saeidnia H.R., Kozak M., Marengo A., Lund B.D., Ausloos M., Ghiasi N. (2024). Principles of digital professionalism for the metaverse in healthcare. BMC Med. Inform. Decis. Mak..

[25] Li Y., Gunasekeran D.V., RaviChandran N., Tan T.F., Ong J.C.L., Thirunavukarasu A.J., Polascik B.W., Habash R., Khaderi K., Ting D.S. (2024). The next generation of healthcare ecosystem in the metaverse. Biomed. J..

[26] Veras M., Labbé D.R., Furlano J., Zakus D., Rutherford D., Pendergast B., Kairy D. (2023). A framework for equitable virtual rehabilitation in the metaverse era: Challenges and opportunities. Front. Rehabil. Sci..

[27] Tretter M., Samhammer D., Ott T., Dabrock P. (2023). Towards an ethics for the healthcare metaverse. J. Metaverse.

[28] Park S.M., Kim Y.G. (2022). A metaverse: Taxonomy, components, applications, and open challenges. IEEE Access.

[29] Bindewari S., Raghav A., Tiwari R. (2025). Introduction of cyber security with metaverse: Challenges and applications. Exploring the Metaverse.

[30] Kim H.Y., Myung S.H., Cho I.Y. (2025). Enhancing nurse-parent partnership for NICU nurses by investigating multi-modal learning with a hybrid simulation approach that integrates metaverses and real-world training. Nurse Educ. Pract..

[31] Bosch-Barceló P., Climent-Sanz C., Martínez-Navarro O., Masbernat-Almenara M., Pakarinen A., Ghosh P.K., Fernández-Lago H. (2024). A treadmill training program in a gamified virtual reality environment combined with transcranial direct current stimulation in Parkinson’s Disease: Study protocol for a randomized controlled trial. PLoS ONE.

[32] Morone G., Tramontano M., Paolucci S., Cerasa A., Ciancarelli I., Cinnera A.M., Iosa M., Calabrò R.S. (2025). Tailoring robot-assisted arm training to individuals with stroke: Bridging neuroscience principles and clinical practice. Front. Neurol..

